# *Planktothrix agardhii* versus *Planktothrix rubescens*: Separation of Ecological Niches and Consequences of Cyanobacterial Dominance in Freshwater

**DOI:** 10.3390/ijerph192214897

**Published:** 2022-11-12

**Authors:** Tomasz Lenard, Małgorzata Poniewozik

**Affiliations:** 1 Department of Animal Physiology and Toxicology, Faculty of Medicine, The John Paul II Catholic University of Lublin, Konstantynów 1I, PL-20-708 Lublin, Poland; 2 Department of Plant Physiology and Biotechnology, Faculty of Medicine, The John Paul II Catholic University of Lublin, Konstantynów 1I, PL-20-708 Lublin, Poland

**Keywords:** *Planktothrix*, vertical distribution of phytoplankton, deep chlorophyll maximum, physicochemical parameters of water, ecological status, PMPL, PSI, Estonian index

## Abstract

Cyanobacteria dominate lakes under diverse trophic conditions. Of these, two harmful filamentous cyanobacterial species, namely *Planktothrix agardhii* and *P. rubescens*, occupy completely different ecological niches in which they can form dense populations. In the present study, we investigated the effects of environmental conditions on the growth and vertical distribution of these species in lakes of different trophic statuses. Moreover, we underscored certain inconveniences in the assessment of the ecological status of lakes according to the European Union Water Framework Directive. The highest biomass of *P. agardhii* was recorded in eutrophic lake at a depth of 0.5–1 m, under high light intensity. Meanwhile, the highest biomass of *P. rubescens*, at which the deep chlorophyll maximum was recorded, was observed in mesotrophic lakes at a depth of 11–12 m, often below the euphotic zone under very low light intensity. *P. rubescens*, but not *P. agardhii*, exerted a strong allelopathic effect on the diversity and biomass of phytoplankton. Moreover, both species utilised different dissolved nitrogen fractions for their growth; specifically, *P. agardhii* used ammonium nitrogen, whilst *P. rubescens* used nitrate nitrogen. Furthermore, dissolved phosphorus produced a potentially limiting effect on *P. rubescens* growth. Overall, the tested Polish PMPL, German PSI, and Estonian phytoplankton indices were indeed useful in the assessment of the ecological status of lakes, albeit limited to the eutrophic lake with a high biomass of cyanobacteria (*P. agardhii*) in the upper water layers. However, problems arose in the proper assessment of lakes with a high biomass of cyanobacteria (*P. rubescens*) with a deep chlorophyll maximum outside the range of the euphotic zone. In such cases, two of the tested indices, namely the Polish and German indices, allowed sample collection from the euphotic layers, which significantly affected the number of samples included in the calculation. Consequently, the correct calculation of the ecological status of the lake was uncertain. Only the Estonian index allowed for a sample collection from two to three thermal layers of water, including the bloom layer of *P. rubescens*. Hence, the Estonian index offered the best fit for calculations.

## 1. Introduction

The ongoing global warming has altered nearly every component of the aquatic environment. The manifestation of these changes is often a rapid rise in air temperature and, consequently, water temperature. This results in abiotic and biotic changes, which disrupt the aquatic ecosystems [1,2,3,4,5]. Only organisms characterised by high ecological plasticity can cope with such changes. The phylogenetically ancient cyanobacteria of the empire of Prokaryota are an example of such organisms [6]. These prokaryotes can accumulate compatible solutes, which help them survive under and acclimate to adverse environmental conditions [7,8]. Hence, these species exhibit great diversity, from unicellular through filamentous to coccoid colonial forms, often enclosed in mucilaginous sheaths that allow them to inhabit almost any environment, from thermal to cold waters, from acidic to alkaline waters, from fresh, brackish, to saline waters, from wastewater to drinking water, and from fertile waters to nutritionally poor ones [7,9,10,11,12,13,14,15]. In every one of these aquatic ecosystems, cyanobacteria can grow rapidly, often forming mass appearances on the water surface called blooms [16], which can be detected visually from changes in water colour and even observed and measured using satellite sensors [17]. However, cyanobacterial blooms reduce light penetration to deeper water layers, thereby negatively affecting the growth of other algae and submerged macrophytes or the success of predators hunting prey [18,19] that may directly affect biogeochemical cycles in aquatic ecosystems [20,21]. Moreover, many cyanobacterial species can produce metabolites that allow them to compete effectively through allelopathy [22]. Unfortunately, amongst these secondary metabolites, cyanotoxins, such as anatoxin-a, microcystins, nodularins, cylindrospermopsin, and saxitoxins, often pose a threat to the entire ecosystem, including aquatic animals, as well as affecting human health [23,24,25]. Thus, every aspect of the mass appearance of cyanobacteria can be considered a cyanobacterial harmful algal bloom (cyanoHAB) [26]. In this context, cyanobacteria are an important phytoplankton group in the assessment of the ecological status of lakes, particularly in relation to the Water Framework Directive (WFD) adopted by the European Union legislation and implemented by its each state member [27,28]. According to the WFD, phytoplankton metrics should consider species composition, species abundance or biomass, and phytoplankton bloom intensity.

The genus *Planktothrix* belongs to the bloom-forming cyanobacteria. In particular, two species of this genus, namely *P. agardhii* (Gomont) Anagnostidis & Komárek and *P. rubescens* (De Candolle ex Gomont) Anagnostidis & Komárek, present highly similar morphological structures. The range of dimensions (length and width) of their solitary and free-floating trichomes often overlaps and apical cells, with or without the calyptra, cause additional problems in their taxonomic distinction. However, differences in their photosynthetic pigment content and stable phycobilin ratio under fluctuating environmental conditions, as well as the prominence of the green pigment phycocyanin (PC) in *P. agardhii* and the red pigment phycoerythrin (PE) in *P. rubescens* cells, may be useful in their identification [29]. Both species are filamentous cyanobacteria devoid of heterocysts but equipped with a group of gas vesicles (aerotopes), which are important to facilitate buoyancy in aquatic environments and allow vertical migration in stagnant waters, such as lakes and reservoirs [30]. This migration ability allows these cyanobacteria to occupy an optimal position in the aquatic environment and follow changing light conditions throughout the day or search for nutrient-rich waters, as required. Thus, both species occupy distinct niches according to light intensity and temperature. Specifically, *P. agardhii* inhabits upper water layers with high light intensity and temperature, which are typical of shallow eutrophic lakes. Conversely, *P. rubescens* develops well in deeper water layers with low light intensity and temperature and is responsible for the phenomenon called the deep chlorophyll maximum (DCM) in mesotrophic lakes [31]. As a result of this niche division, these species may respond differently to high light intensity, as manifested by more frequent photoinhibition in *P. rubescens* than in *P. agardhii* [32,33]. Based on these ecological features, the species are treated differently with respect to the functional groups of phytoplankton, with *P. agardhii* being included in group S1 and *P. rubescens* in group R [34,35,36]. Nevertheless, genomic data suggest that these two green/red species should be considered an ecotype occupying different ecological niches [37,38]. Interestingly, however, these species do not occur simultaneously, which may be the result of their allelopathic interactions, as previously reported by Oberhaus et al. [39]. Moreover, the mass appearance of *Planktothrix* species is most often associated with the occurrence of toxins in water, mainly microcystins, in addition to many secondary metabolites, which can affect the functioning of the entire ecosystem [40,41,42,43,44]. The blooms of *P. agardhii* are globally distributed and have been recognised as a direct consequence of the eutrophication often caused by discharge of agricultural or urban wastewater [45,46,47]. In contrast, the development of *P. rubescens* in metalimnion of deep clearwater lakes is partially attributed to climate warming, stability of water column and reduced of water mixing [48,49,50]. With reference to all these characteristics, both species are similar, yet different in many ways. Hence, they are interesting objects of study.

To this end, the aims of the present study were to (i) determine the effects of environmental conditions on the occurrence of filamentous cyanobacterial blooms in lakes with different trophic statuses, (ii) determine the vertical distribution and dynamics of blooms of two different species of the genus *Planktothrix*, which pose a potential threat to the aquatic ecosystems and human health, and (iii) underscore certain inconveniences in the assessment methods of the ecological status of water based on phytoplankton indices according to the WFD.

## 2. Materials and Methods

The present study was conducted in two lakes, namely Glinki (51°50′ N 23°55′ E, 157.4 m a.s.l) and Piaseczno (51°23′ N, 23°01′ E, 170.6 m a.s.l), located on the Łęczna–Włodawa Plain in eastern Poland, which is part of the large cross-border Polesie region [51]. The lakes markedly differ in terms of hydrological and morphological features. Lake Glinki is small, shallow, dimictic, and eutrophic, whereas Lake Piaseczno is two times larger, deep, dimictic, and mesotrophic (Table 1). Additionally, Lake Glinki has inflow and outflow of the Tarasienka River in the southeastern part of the lake, whereas Lake Piaseczno is devoid of inflows and outflows [52,53]. However, despite increasing precipitation trends, the amplitude of water level fluctuations in this lake is very high, reaching 1.68 m in the last 20 years. Meanwhile, the water level in Lake Piaseczno is estimated to decrease at a rate of approximately 1 cm per year, which is related to a decrease in water resources in the chalk layer of the Lublin Upland and changes in the direction of underground outflow from the catchment area of the upper Wieprz River [54].

The phytoplankton biomass, chlorophyll-*a* concentration, and physicochemical parameters of water were analysed during thermal stratification from May to September 2012 (Lake Glinki) and 2014 (Lake Piaseczno). Water samples for analyses were collected monthly from the deepest part of the lakes using a Ruttner-type water sampler (capacity = 2.0 L). All water samples from both lakes were collected from the depth of 0.5 m, followed by 1 and 6 m in Lake Glinki or from 1 and 12 m in Lake Piaseczno, at 1 m intervals. Therefore, we collected 7 and 13 samples each month, totalling 35 and 65 samples for lakes Glinki and Piaseczno, respectively. In the laboratory, the samples were analysed using spectrophotometric methods to determine the concentration of chlorophyll-*a* [55], total phosphorus (TP), total nitrogen (TN), inorganic phosphorus (P–PO_4_), and inorganic nitrogen (N–NO_3_ and N–NH_4_) [56]. The samples for phytoplankton analyses were fixed with Lugol’s iodine solution and a formalin glycerine mixture. Phytoplankton abundance was determined according to the standard Utermöhl’s method [57], and algal biovolume was calculated using the formula described by Hillebrand et al. [58]. The water samples were transferred to a settling chamber with a 5–50 mL capacity. After sedimentation, the algal abundance was evaluated using an inverted microscope (Zeiss Axiovert 135). In each chamber, small phytoplankton species were counted on the belts, whereas larger forms (filamentous or coccal colonies) were counted on the entire bottom of the chamber. The unit length of 100 µm and a surface of 300 µm^2^ were considered to be one individual for filamentous and coccal colonies, respectively. Additionally, the samples for taxonomic analyses of phytoplankton were collected using a plankton net (20 µm mesh size) and were left without fixation to observe live specimens under a light microscope (Nikon Eclipse 80i). All samples were identified to the species level, if possible. To determine the differences in the phytoplankton species composition, the Shannon–Wiener diversity index [59] and Pielou’s evenness index [60] were calculated based on the abundance of the phytoplankton community. Other physicochemical parameters, such as water temperature [to calculate the range of the mixing zone (Z_mix_)—defined as the deepest water layer to which the temperature gradient was <1 °C m^−1^], electrolytic conductivity (EC), pH values, and water transparency, as measured with a Secchi disc visibility (SD), were analysed in situ. Additionally, to precisely calculate the range of the euphotic zone (Z_eu_), photosynthetic active radiation (PAR) was measured using the Li-Cor 192SA underwater quantum flat meter. The correlations between abiotic (physicochemical) and biotic (the concentration of chlorophyll-*a*, phyoplankton biomass) parameters, were evaluated with Spearman’s rank correlation test. All calculations were performed with Statistica 13 package.

To assess the ecological status of lakes according to the WFD, we used the multi-metric phytoplankton index for Polish lakes (PMPL) proposed by Hutorowicz and Pasztaleniec [61]. According to these criteria, both lakes were assumed to be stratified and belong to type 7a—lowland stratified lakes of the Lake District in the Polesie sub-province [62]. The final PMPL index for ecological quality (EQ) ranges from 0 (best status) to 5 (worst status). Nevertheless, to verify the accuracy of the PMPL index, we also adopted the multi-metric index proposed by Mischke et al. [63] for phytoplankton-based lake assessment in Germany (PSI). We adopted the PSI metric in our investigation because it considers a list of indicator taxa with their trophic scores as well as weighting factors that are important in the case of changes in phytoplankton community between the studied lakes. The German phytoplankton assessment system for lakes is based on a combination of typological criteria from the WFD and lake types proposed by LAWA [64]. According to these criteria, Lake Glinki was classified as type 13—a lowland stratified lake with a ratio of lake volume to catchment area (VQ) of <1.5—whereas Lake Piaseczno was classified as type 10—a lowland stratified lake with VQ of >1.5 and <15. The PSI index ranges from 0.5 to 5.5, where 0.5 indicates the best status and 5.5 the worst status. These values correspond to the five ecological status classes (1 to 5) and can be interpreted as EQ. In the present study, we also tested the Estonian index (EI), which allows for sampling of two to three thermal layers of water, including the epi-, meta-, and hypolimnion, which may be important in the case of *P. rubescens* blooms in the metalimnion. The EI involves four metrics [65,66]. Value of a single metric ranges from 1 to 5, and in the final score, each parameter has an equal weight. The arithmetical average of each parameter value yields the final lake phytoplankton score, which is achieved by rounding off. The results of EQ (ranging between 0 and 5) calculated based on PMPL, PSI, and EI were transformed to the normalised values of the ecological quality ratio (EQR), which ranges between 0 and 1 and can be divided into a five-grade classification system: 0–0.2, bad; 0.21–0.4, poor; 0.41–0.6, moderate; 0.61–0.8, good; and 0.81–1, high ecological status.

## 3. Results

### 3.1. Biological and Physical Characteristics of the Studied Lakes

**Figure 1:**
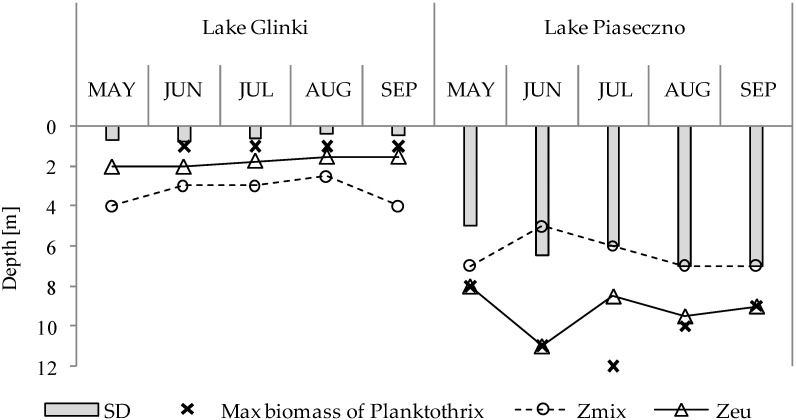
Variation in Secchi disk visibility (SD), the range of the euphotic zone (Zeu), the mixing zone (Zmix), and the depths with maximum *Planktothrix agardhii* and Planktothrix rubescens biomass during the vegetative season in Lake Glinki and in Lake Piaseczno, respectively.

In the studied lakes, strong vertical variability in the biological and physical parameters was recorded. Water transparency (SD) in Lake Glinki was very low and reached a maximum of 0.8 m coupled with a small Z_eu_ of approximately 2 m, whereas Z_mix_ was deeper throughout the season, ranging from 3 to 4 m (Figure 1). Conditions in Lake Piaseczno were much better; as such, SD was never below 5 m, the boundary of Z_eu_ reached 11 m, and Z_mix_ never exceeded 7 m. These light and mixing regimes significantly affected the presence of cyanobacterial blooms with dominant species of the genus *Planktothrix*, specifically *P. agardhii* in Lake Glinki and *P. rubescens* in Lake Piaseczno. The biomass of both species showed high vertical variability. The highest biomass of *P. agardhii* was always noted in the upper layer of Z_eu_ and Z_mix_, whereas the highest biomass of *P. rubescens* was at the edge or even below Z_eu_ and far below the range of Z_mix_ (Figure 1).

**Figure 2:**
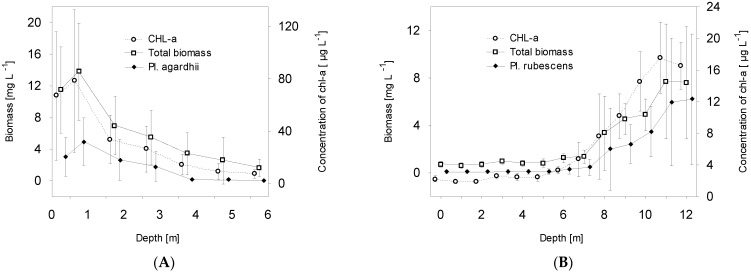

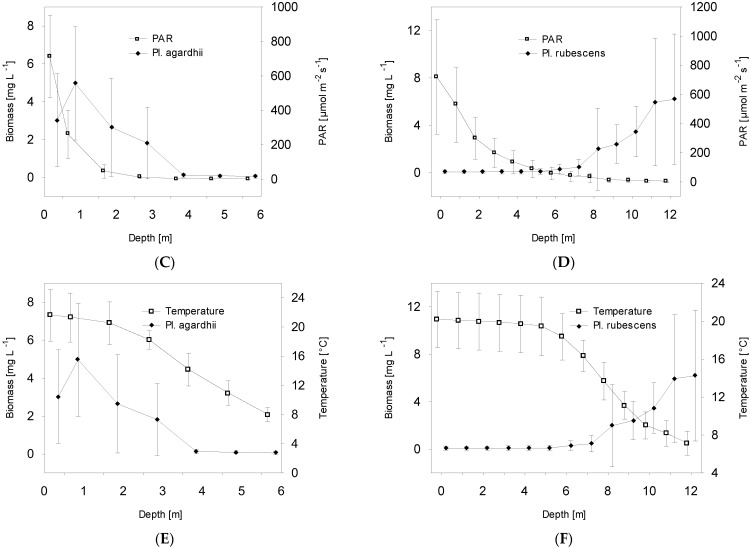
Mean values (±standard deviations) of selected biological and physical parameters of water in Lake Glinki (**A**,**C**,**E**) and Lake Piaseczno (**B**,**D**,**F**). Explanations: CHL-*a*, concentration of chlorophyll-a; PAR, photosynthetically active radiation.

**Figure 3:**
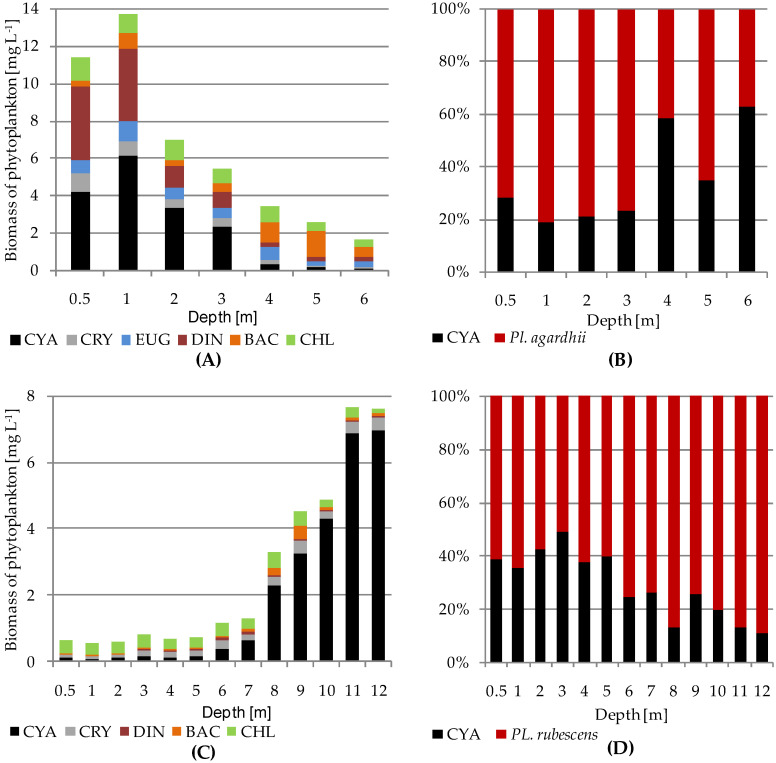
Vertical variation of mean values of the phytoplankton biomass and percentage shares of the genus *Planktothrix* in the Cyanoprokaryota group in Lake Glinki (**A**,**B**) and in Lake Piaseczno (**C**,**D**). Explanations: CYA, Cyanoprokaryota; CRY, Cryptophyceae; EUG, Euglenophyceae; DIN, Dinophyceae; BAC, Bacillariophyceae; CHL, Chlorophyta.

**Figure 4:**
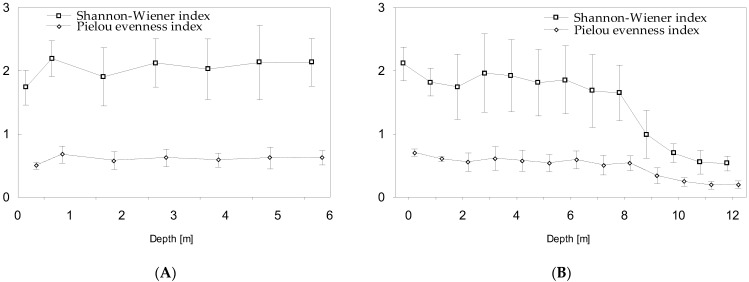
Mean values (±standard deviations) of Shannon–Wiener diversity index and Pielou evenness index in Lake Glinki (**A**) and Lake Piaseczno (**B**).

The presence of *P. agardhii* in Lake Glinki was recorded mainly to the depth of 3 m; however, the highest mean value of its biomass during the vegetative season (4.96 mg L^−1^) was noted at a depth of 1 m, with the highest mean value of chlorophyll-*a* concentration (79.84 µg L^−1^), high light intensity expressed as PAR exceeding 200 µmol m^−2^ s^−1^, and mean water temperature exceeding 20 °C (Figure 2A,C,E). Interestingly, the biomass of *P. agardhii* in the upper water column (0.5–3 m), which shares in the cyanoprokaryota group, exceeded 70% and was coupled with the high biomass of dinoflagellates (mean value at 1 m–3.88 mg L^−1^) dominated by *Ceratium hirundinella* and the frequent but not abundant presence of other phytoplankton groups (Figure 3A,B). Nevertheless, in the lower water column (4–6 m), the mean value of *P. agardhii* biomass was negligible, and other phytoplankton groups, such as Bacillariophyceae, dominated, albeit with smaller biomass values (Figure 3A). In contrast, significant biomass of *P. rubescens* in Lake Piaseczno was recorded in the metalimnion at a depth of 8–12 m, and it was almost absent in the upper water column up to the depth of 5 m or accounted for a greater share in phytoplankton but with a rather small biomass at a depth of 6–7 m. Of note, in the upper water layers (0.5–7 m), the mean values of total phytoplankton biomass during the vegetative season were low (0.5–1.3 mg L^−1^), whilst in the lower water layers with cyanobacterial blooms (8–12 m), they were at least several times higher, ranging from 3.3 to 7.6 mg L^−1^ (Figure 2B and Figure 3C). Cyanobacterial biomass in the metalimnion was completely dominated by *P. rubescens*, often exceeding 80% of the biomass of the group (Figure 3D). Importantly, the presence of *P. rubescens* strongly affected the total phytoplankton biomass, resulting in a DCM in the metalimnion of Lake Piaseczno (Figure 2B). The highest mean *P. rubescens* biomass was noted at a depth of 11–12 m (~6.5 mg L^−1^), with mean chlorophyll-*a* concentration of approximately 17 µg L^−1^, extremely low values of PAR (~4 µmol m^−2^ s^−1^), and mean water temperature of around 8 °C (Figure 2D,F). The relationships between *P. agardhii* or *P. rubescens* biomass and the parameters described above were highly statistically significant. Specifically, *P. agardhii* biomass was positively correlated with PAR, water temperature, chlorophyll-*a* concentration, and total phytoplankton biomass, whereas *P. rubescens* was negatively correlated with PAR and water temperature but positively correlated with chlorophyll-*a* concentration and total phytoplankton biomass (Table 2). However, different effects of these species on phytoplankton diversity were observed. Under the dominance of *P. agardhii* in Lake Glinki, the values of Shannon–Wiener diversity index (H′) and Pielou evenness index (P) were stable throughout the water column or only slightly higher at a depth of 1 m, where the biomass of *P. agardhii* was the highest (Figure 4A). Moreover, due to the frequent presence of species from other phytoplankton groups, the phytoplankton community was more diverse (Figure 3A). Hence, the effect of *P. agardhii* on phytoplankton community composition was statistically insignificant (Table 2). The opposite effect was observed for *P. rubescens* biomass in Lake Piaseczno. In the upper water column up to the depth of 7 m, the mean values of diversity indices were similar to those noted in Lake Glinki; however, at depths where *P. rubescens* was dominant in the metalimnion, the H′ and P indices sharply declined to values below 1 and ~0.2, respectively (Figure 4B).

### 3.2. Physicochemical Characteristics of the Studied Lakes

**Figure 5:**
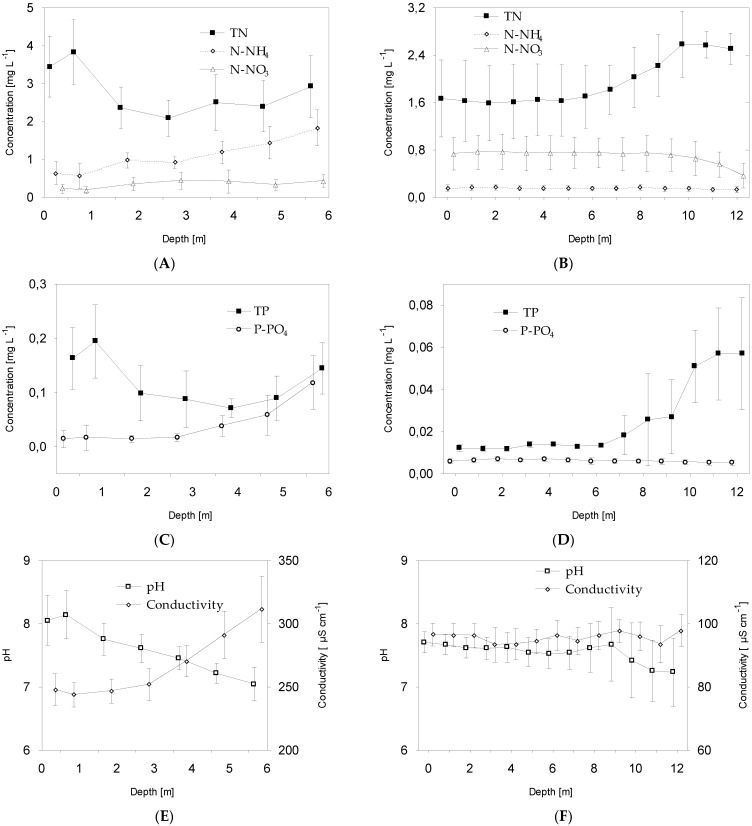
Mean values (±standard deviations) of selected chemical and physicochemical parameters of water in Lake Glinki (**A**,**C**,**E**) and Lake Piaseczno (**B**,**D**,**F**). Explanations: TN, total nitrogen; N-NO_3_, nitrate nitrogen; N-NH_4_, ammonium nitrogen; P-PO_4_, phosphate phosphorus; TP, total phosphorus.

The total (TN and TP) and dissolved (N-NH_4_, N-NO_3_, and P-PO_4_) fractions of biogenic compounds in water were significantly correlated with the biomass of *P. agardhii* and *P. rubescens* (Table 2). In addition, the relationship trends were similar for both species. The highest mean values of TN and TP were always noted at the depth with the highest biomass of *Planktothrix* species. For instance, in Lake Glinki, at a depth of 1 m, the mean values of TN and TP reached respectively 3.7 and 0.19 mg L^−1^, whereas in Lake Piaseczno, at a depth of 10–12 m, these values reached respectively 2.4 and 0.055 mg L^−1^ (Figure 5A–D). The higher values of the total fractions of nutrients corresponded to the values of the dissolved fractions, whose concentrations were always much higher in Lake Glinki, confirming its eutrophic nature. Specifically, the concentrations of N-NH_4_ and P-PO_4_ were very high in Lake Glinki, reaching the highest mean values at a depth of 6 m (N-NH_4_ = 1.8 mg L^−1^, and P-PO_4_ = 0.12 mg L^−1^) and the lowest values at the depth at which the phytoplankton biomass was the highest (Figure 5A,C). Reuse of these N and P fractions by *P. agardhii* was significant (r = −0.38 for N-NH_4_ and r = −0.41 for P-PO_4_, *p* < 0.05, Table 2). A similar significant decreasing trend of dissolved nutrient concentrations (N-NO_3_ and P-PO_4_) was observed in the metalimnion of Lake Piaseczno (r = −0.55 for N-NO_3_, and r = −0.38 for P-PO_4_, *p* < 0.001, Table 2), confirming the incorporation of these fractions into *P. rubescens* biomass (Figure 5B,D). Interestingly, *P. agardhii* biomass in Lake Ginki was strongly and positively correlated with pH but negatively correlated with EC, whereas these correlations were not observed for *P. rubescens* biomass in Lake Piaseczno (Figure 5E,F and Table 2).

### 3.3. Assessment of Ecological Status of the Studied Lakes Based on Phytoplankton Indices

**Figure 6:**
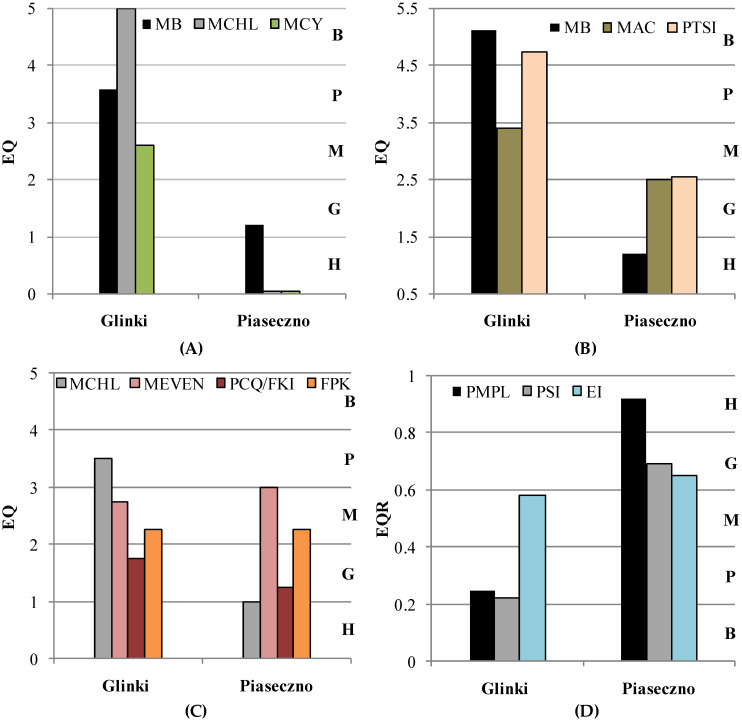
Ecological Quality (EQ) values and status class assessment according to: (**A**) Polish metrics of lakes ecological status assessment. Abbreviations: H, high; G, good; M, moderate; P, poor; B, bad ecological status; MB, metric of total biomass of phytoplankton; MCHL, metric of chl-*a* concentration; MCY, metric of biomass of cyanobacteria; (**B**) German metrics of lakes ecological status assessment. Abbreviation: MB, metric of biomass of phytoplankton; MAC, metric of algal classes’ PTSI, metric phytoplankton taxa seen index; (**C**) Estonian metrics of lakes ecological status assessment. Abbreviation: MCHL, metric of chl-*a* concentration; MEVE, metric of evenness of phytoplankton species; PCQ/FKI, metric of Nygaard’s modified compound quotient; FPK, metric of description of the community. (**D**) Ecological quality ratio (EQR) based on PMPL, PSI, and EI indices calculated for the studied lakes. Abbreviations: PMPL, index for Polish lakes; PSI, index for German lakes; EI–index for Estonian lakes.

The assessment of the studied lakes’ ecological status using the three indices (PMPL, PSI, and EI) showed some discrepancies. In particular, PMPL and PSI, composed of three different metrics, recognised Lake Glinki as being in a moderate, poor, or even bad condition (Figure 6A,B); however, based on the final calculations of EQR, Lake Glinki was placed in the same poor ecological status according to PMPL and PSI (Figure 6D). Next, EI, composed of different criteria, including specific analysis of the phytoplankton community using four metrics, yielded unexpected results. Based on the chlorophyll-*a* metric (MCHL), Lake Glinki was recognised as being in a poor condition; however, based on the description of the community (FPK) and Nygard’s modified compound quotient (PCQ/FKI) metrics, the lake was in a moderate or even good condition. The final EQR calculation showed a moderate (almost good) ecological status of Lake Glinki (Figure 6C,D). The assessment of the ecological status of Lake Piaseczno was much better. Despite the presence of *P. rubescens* blooms in the metalimnion, PMPL placed this lake at a high ecological status (Figure 6D). The extremely low values of the MCHL and MCY (biomass of cyanobacteria) metrics affected the final score the most (Figure 6A). According to PSI and EI, Lake Piaseczno was placed in a good ecological status (Figure 6D). Nevertheless, two metrics of PSI, namely MAC (algal classes) and PTSI (phytoplankton taxa seen index), placed this lake at the boundary between good and moderate statuses. Meanwhile, the third metric, biomass (MB), indicated a high ecological status of Lake Piaseczno and affected the final score the most. Unexpectedly, two metrics of EI, namely MEVEN (evenness) and FPK (description of the community), recognised Piaseczno Lake as being in a moderate or moderate/poor condition (Figure 6C). Nevertheless, the final score based on EQR indicated this lake as having good ecological status according to EI and PSI (Figure 6D).

## 4. Discussion

In the present study, we analysed the dominance of two species, namely *P. agardhii* and *P. rubescens*, which inhabit radically different ecological niches. *P. agardhii* blooms have been frequently detected in different regions of Poland [67]; however, the mass appearance of *P. rubescens*, which is common in alpine or glacial lakes [34,68] or in the metalimnion waters of temperate regions [69], is rare in Poland and has been detected exclusively in lakes on the Łęczna–Włodawa Plain [70,71,72]. Hence, we selected two different lakes from this region of Poland, namely lakes Glinki and Piaseczno, which allowed us to compare the blooms of these two cyanobacteria developing under similar climatic conditions.

One of the most important environmental factors enabling the development of autotrophic species in water is solar radiation, particularly in the visible light range of 400–700 nm, which constitutes PAR that phytoplanktonic organisms can use for photosynthesis. However, phytoplanktonic species can absorb light in different spectra using appropriate photosynthetic pigments. The green-pigmented *P. agardhii* developed well in the upper water column of Lake Glinki at a depth of 0.5–3 m, although the highest value of its biomass was generally detected just below the water surface at a depth of 0.5–1 m, where both light intensity (mean PAR = ~200–800 µmol m^−2^ s^−1^) and water temperature (>20 °C) were high. In contrast, the red-pigmented *P. rubescens* developed well at a depth of 8–12 m, and its highest biomass was recorded at a depth of 11–12 m, where light intensity (mean PAR = ~4 µmol m^−2^ s^−1^) and water temperature (~8 °C) were extremely low. The occurrence of these two species under such extremely different light intensities has been observed both under laboratory [33,73] and natural [32,50,68,74,75,76] conditions. Owing to the presence of phycoerythrin in cells, *P. rubescens* often develops in shaded layers of water, which renders it more sensitive to high light intensity or rapid changes in light than *P. agardhii*. However, recent studies have shown that both *Planktothrix* species have evolved a photoprotective mechanism triggered by the photoactive orange protein carotenoid. Nevertheless, this mechanism works faster in *P. agardhii*, which renders it more resistant to high light intensity and allows for faster fluorescence recovery; however, this mechanism is much less effective in *P. rubescens* [77]. To counteract photoinhibition, *P. rubescens* exploits the density gradient of water and develops DCM at a low light intensity and water temperature in the metalimnion [32,33,68,74]. The increasing of water transparency and decreasing lake size seem to be crucial for developing DCM in lakes [78]. Additionally, the depth of DCM layer deepens with an increase in lake clarity and a decrease in light attenuation, and the thickness of the DCM layer is dependent on the lake size [31]. Hence, the correct localization of the depth and thickness of DCM layer has a significant impact on the correct assessment of the ecological status of deep lakes. In Lake Piaseczno, the *P. rubescens* population developed in the metalimnion (8–12 m) was far from the range of Z_mix_ (reaching max to the depth of 7 m), supporting the stability of environmental conditions. However, recent studies have indicated that in stratified lakes, any disturbances in the annual stratification pattern can negatively affect the growth success of *P. rubescens* [50]. Hence, the mass appearance of *P. rubescens* in Lake Glinki is unlikely because the extent of the mixing zone reaches almost half of the maximum depth of this lake, and complete darkness is present in the deeper water layers. Under such conditions, *P. rubescens* should migrate to the upper water layers, where it can be exposed to mixing conditions, high light intensity, and, consequently, photoinhibition. Interestingly, however, to develop in the metalimnion of Lake Piaseczno, *P. rubescens* must have an adequate amount of light. Hence, a very low total biomass of phytoplankton and low concentration of chlorophyll-*a* was detected in the upper water layers of this lake (0.5–7 m), resulting in a high water transparency at 7 m. This raises the question of whether *P. rubescens* inhibits the growth of other algae and thus ensures adequate light intensity in the metalimnion. In the present study, we noted a significant decline in phytoplankton diversity coupled with an increase in *P. rubescens* biomass in the metalimnion and an increase in the diversity in the upper water layers of Lake Piaseczno (0.5–7 m) coupled with several times lower values of total phytoplankton biomass (Figure 3C,D and Figure 4B), which was statistically confirmed (Spearman’s rank correlation for H′, P, and number of species always above −0.6, and for total biomass 0.85, *p* < 0.001, Table 2). This is consistent with previous experimental findings, which suggested the presence of certain *P. rubescens*-produced allelopathic inhibitory substances other than microcystins acting against algae [39]. Furthermore, the observed decline in biodiversity was not ascertained in the presence of *P. agardhii* in Lake Glinki, where this cyanobacterial species often co-existed with a similar biomass of the dinoflagellate *Ceratium hirundinella*, suggesting a weak or lack of allelopathic effect of this species on other algae. These findings may be partially supported by experimental results showing a reduction in growth and alterations in the morphology of *P. agardhii* in co-cultures with *Microcystis aeruginosa* [78] or *Aphanizomenon gracile* [79]. Additionally, another study showed suppression of *P. agardhii* growth in the presence of extracts obtained from *P. rubescens* cultures [39], which may explain the lack of simultaneous dominance of the two species.

Interesting results were obtained regarding available biogenic compounds. The present study showed variability in chemical factors amongst the studied lakes. This was mainly reflected in the pool of dissolved nitrogen compounds available to the phytoplankton community. Lake Glinki recorded higher concentrations of ammonium nitrogen (N-NH_4_), whereas Lake Piaseczno showed higher concentrations of nitrate nitrogen (N-NO_3_). In Lake Glinki, the highest concentration of N-NH_4_ was recorded at a depth of 6 m, although this may have been the effect of the proximity of bottom sediments (max. depth of the lake ca. 8.8 m). Nevertheless, high concentrations of this nitrogen fraction were maintained throughout the water column, except in the layer with high *P. agardhii* biomass (0.5–1 m), where a significant decrease in N-NH_4_ concentration was recorded. Meanwhile, N-NH_4_ concentration in Lake Piaseczno was very low and was not correlated to *P. rubescens* biomass. However, N-NO_3_ concentration in Lake Piaseczno was high, although it was significantly decreased in layers dominated by *P. rubescens*, as confirmed based on a strong and significant correlation (Table 2). Neither species forms heterocysts or can fix molecular nitrogen (N_2_) from the air [29], suggesting that both species utilised different dissolved nitrogen fractions for their growth. Specifically, *P. agardhii* used ammonium nitrogen, whilst *P. rubescens* used nitrate nitrogen. These results are consistent with previous findings, indicating an important role of *P. agardhii* in the recycling of N-NH_4_ from water [47]. Moreover, N-NH_4_ incorporation lent *P. agardhii* some independence from the nitrogen pool, which may explain its co-occurrence with other phytoplankton species in Lake Glinki. In contrast, as a result of the high allelopathic activity of *P. rubescens* and its complete dominance in the metalimnion of Lake Piaseczno, the entire N-NO_3_ fraction was almost exclusively available for this species. In both lakes, decreases in the concentrations of dissolved nitrogen compounds were correlated with increases in the biomass of *Planktothrix* species and concentration of TN, confirming the incorporation of dissolved compounds from water into the biomass of these cyanobacteria. The concentrations of dissolved and total fractions of phosphorus were always higher in Lake Glinki and much lower in Lake Piaseczno, confirming the eutrophic and mesotrophic statuses of these lakes, respectively. Moreover, in Lake Glinki, the concentration of both fractions increased with depth, reaching the highest values at a depth of 6 m, where phytoplankton biomass was very low. Therefore, the elevated concentration of dissolved phosphorus at greater depths may be attributed to the proximity of bottom sediments, similar to that for dissolved nitrogen fractions. However, the biomass of both *Planktothrix* species was significantly correlated with both phosphorus fractions (Table 2); as such, the concentration of phosphate phosphorus (P-PO_4_) was often very low (4–7 µg L^−1^), and that of TP was high (ca. 55 µg L^−1^) in the metalimnion of Lake Piaseczno, suggesting the limitation of *P. rubescens* growth by dissolved inorganic phosphorus. Evidently, however, under depleted dissolved inorganic phosphorus conditions, *P. rubescens* can avoid this limitation by exploiting dissolved organic phosphorus or other sources of phosphorus received from the remineralisation of organic matter by heterotrophic bacteria [75,80,81]. Notably, changes in physicochemical conditions were significant only in Lake Glinki. The increase in EC in the lower water column was probably related to the increase in dissolved nitrogen and phosphorus content due to the proximity of bottom sediments. Nevertheless, the decrease in EC and increase in pH at depths where *P. agardhii* biomass was high may be attributed to the effect of the incorporation of dissolved nutrients into phytoplankton biomass during intensive photosynthesis under high light irradiance. This effect was not observed for *P. rubescens* biomass.

Furthermore, the present study confirmed that lakes in which *Planktothrix* species developed varied in terms of their trophic status. In particular, *P. agardhii* grew in waters with high nutrient concentrations and low water transparency and co-existed with diverse phytoplankton communities, confirming the eutrophic status of Lake Glinki. In contrast, *P. rubescens* grew in a deep lake with high water transparency, low nutrient concentrations, and low phytoplankton diversity, particularly in the bloom layer, confirming the mesotrophic status of Lake Piaseczno. However, in the context of the WFD, the assessment of the studied lakes according to the selected phytoplankton indices (Polish: PMPL; German: PSI; and Estonian: EI) yielded inconsistent results. Specifically, PMPL and PSI allow for water sampling using two approaches, considering only the epilimnion or the entire euphotic layer of deep lakes [61,63]. In EI, sampling depends on the existence of water stratification, and if stratification is present, each layer (epi-, meta-, and hypolimnion) must be sampled [58]. Moreover, PMPL and PSI are based on a similar approach, in which the biomass of phytoplankton and the presence of cyanobacteria play an important role in the calculation of the ecological status of a lake. However, PSI introduces some metrics based on indicator taxa. Conversely, EI follows a different approach, with emphasis on not only phytoplankton biomass, as expressed by chlorophyll-*a* concentration, but also species diversity and the share of flagellate species in the phytoplankton community. In both studied lakes, high biomasses of cyanobacteria of the genus *Planktothrix* were recorded, with generally low trophic values [34,35,63]. Hence, the studied lakes with cyanobacterial dominance were not expected to achieve a good ecological status, which the EU member states should aim to achieve for surface water [27]. According to the tested indices (PMPL, PSI, and EI), a moderate or poor ecological status was assigned to Lake Glinki, whereas Lake Piaseczno was classified as having good (PSI and EI) or even high (PMPL) ecological status (Figure 6D). These results are interesting because in both studied lakes, the biomass of cyanobacteria was high (two times higher in the metalimnion of Lake Piaseczno than in the epilimnion of Lake Glinki). However, this observation can be explained as follows. The water samples collected to assess the ecological status of Lake Piaseczno based on PMPL and PSI did not include some samples from the bloom layer of *P. rubescens*, which were outside the euphotic zone. Hence, the values of MCY (cyanobacteria) and MCHL (chlorophyll-*a*) metrics were extremely low and affected the final result of PMPL. This suggests that in the presence of cyanobacterial blooms beyond the euphotic layer, both PMPL and PSI may become insensitive. This is in agreement with recent studies that showed that there may be different types of lakes with different localisation of DCM. In some types of lake, DCM may develop on the bottom of the euphotic zone and above the thermocline, in other types of lake, the euphotic zone may reach beyond the thermocline, in which DCM may develop, and the last type is when the euphotic zone is much greater than the thermocline depth and DCM may develop below the thermocline [82]. Hence, the final assessment may overestimate the ecological status of such lakes as Piaseczno, in which the DCM developed below the euphotic layer. Interestingly, EI, which included samples from the bloom layer of *P. rubescens*, also assigned Lake Piaseczno a good ecological status (EQR = 0.65); however, this score was very close to the boundary of good and moderate status, and the result may be attributed to moderate/poor or moderate values evenness and FPK metrics, respectively. Unexpectedly, EI indicated a moderate (EQR = 0.58) but very close to a good ecological status for Lake Glinki. This result was the most affected by the evenness and PCK/FKI metrics, emphasising the presence of flagellate species, specifically *Ceratium hirundinella*, with high biomass in the upper water layers of Lake Glinki (Figure 3A).

Of note, both *P. agardhii* and *P. rubescens* can produce microcystins and other secondary metabolites with a potential toxicity risk for living organisms, including humans [40,41,42,44,83,84,85]. Hence, assessments of the ecological status of lakes should consider the risks of potentially toxic species inhabiting the deeper water layers. If such species occur below the euphotic layer, some phytoplankton indices implemented by individual EU countries, which do not take these water layers into account during calculations [28], may underestimate or overestimate the ecological status of waters, as evidenced in calculations based on PMPL and PSI in the present study.

## 5. Conclusions

1. The development of *Planktothrix* species is closely related to light conditions. In the studied vegetative periods, the highest biomass of *P. agardhii* was recorded at a depth of 0.5–1 m under high light intensity, whilst the highest biomass of *P. rubescens* was recorded at a depth of 11–12 m under very low light intensity, below the boundary of the euphotic layer.
2. *P. rubescens* development led to a rapid and marked decline in biodiversity in the bloom layer (metalimnion) and decreased total phytoplankton biomass in the upper water layers. This implies a significant allelopathic effect of *P. rubescens* on phytoplankton communities. This effect was not observed for *P. agardhii*.
3. *P. rubescens* and *P. agardhii* utilised different dissolved nitrogen fractions for their growth. In particular, *P. agardhii* in the eutrophic Lake Glinki used ammonium nitrogen, whereas *P. rubescens* in the mesotrophic Lake Piaseczno used nitrate nitrogen.
4. Very low values of dissolved phosphorus fraction were recorded in the bloom layer of *P. rubescens*, which may produce a potentially limiting effect on the growth of this species. No such effect was observed for *P. agardhii*.
5. PMPL, PSI, and EI are indeed useful for assessing the ecological status of shallow eutrophic lakes, in which phytoplankton blooms occur in the upper water layers. However, problems arise when phytoplankton blooms occur with DCM in the metalimnion of deep lakes. For instance, the highest biomass of *P. rubescens* occurred outside the euphotic zone. This resulted in a part of the bloom layer being excluded from final calculations based on PMPL and PSI, making it impossible to accurately calculate the ecological status of the lake. Overall, EI, which considers two to three thermal layers of water (depending on the thermal characteristics of a lake), including the phytoplankton bloom layer with DCM, appeared to be the best assessment method.

## Figures and Tables

**Table 1:** General morphological data of the studied lakes [53].

Parameters	Lake Glinki	Lake Piaseczno
Area (ha)	46.9	84.7
Maximum depth (m)	8.8	38.8
Mean depth (m)	2.8	12.6
Mean slope inclination	1° 35′	4° 50′
Maximum length (m)	1031	1464
Maximum width (m)	652	819
Mean width (m)	455	579
Length of shoreline (m)	3018	3788
Volume of lake (thousands m^3^)	1342	10,674
Type of stratification	stratified	stratified
Type of water mixing	dimictic	dimictic
Catchment area (ha)	159.7	284.9

**Table 2:** Spearman’s rank correlation matrix (r) of *Planktothrix agardhii* biomass in Lake Glinki (*n* = 35) and *Planktothrix rubescens* biomass in Lake Piaseczno (*n* = 65) and selected environmental parameters (bold numerals for *p* < 0.001; italic numerals for *p* < 0.05).

Parameters	The Biomass of	
	*P. agardhii*	*P. rubescens*
Depth	**−0.55**	**0.76**
Total nitrogen (TN)	*0.42*	*0.27*
Ammonium nitrogen(N-NH_4_)	−*0.38*	−0.19
Nitrate nitrogen (N-NO_3_)	−0.25	**−0.55**
Phosphate phosphorus (P-PO_4_)	−*0.41*	**−0.38**
Total phosphorus (TP)	**0.57**	**0.71**
pH	**0.54**	0.23
Electrolytic conductivity	−*0.50*	−0.09
Photosynthetic active radiation (PAR)	*0.41*	**−0.68**
Temperature of water	**0.58**	**−0.87**
Concentration of chlorophyll-*a*	**0.79**	**0.72**
Total biomass of phytoplankton	**0.78**	**0.85**
Shannon–Wiener diversity index	0.08	**−0.72**
Pielou evenness index	0.21	**−0.64**
Number of species	−0.25	**−0.71**

## Data Availability

Not applicable.
